# L-Cysteine Alleviates Myenteric Neuron Injury Induced by Intestinal Ischemia/Reperfusion *via* Inhibitin the Macrophage NLRP3-IL-1β Pathway

**DOI:** 10.3389/fphar.2022.899169

**Published:** 2022-06-08

**Authors:** Yifei Gao, Haojie Zhang, Yujin Wang, Ting Han, Jing Jin, Jingxin Li, Yan Tang, Chuanyong Liu

**Affiliations:** ^1^ Department of Physiology, School of Basic Medical Sciences, Cheeloo Medical College, Shandong University, Jinan, China; ^2^ Department of Gastroenterology, Qilu Hospital (Qingdao), Cheeloo College of Medicine, Shandong University, Qingdao, China; ^3^ Department of TCM Rheumatology, China-Japan Friendship Hospital, Beijing, China; ^4^ Provincial Key Lab of Mental Disorders, Shandong University, Jinan, China

**Keywords:** ischemia/reperfusion, L-cysteine, myenteric neuron, macrophages, IL-1β, enteric neural precursor cells

## Abstract

Ischemia/reperfusion injury is a common pathophysiological process in the clinic. It causes various injuries, multiple organ dysfunction, and even death. There are several possible mechanisms about ischemia/reperfusion injury, but the influence on intestinal myenteric neurons and the underlying mechanism are still unclear. C57BL6/J mice were used to establish the ischemia/reperfusion model *in vivo*. Peritoneal macrophages were used for ATP depletion and hypoxia/reoxygenation experiment *in vitro*. L-cysteine, as the substrate of hydrogen sulfide, is involved in many physiological and pathological processes, including inflammation, metabolism, neuroprotection, and vasodilation. In the current study, we confirmed that intestinal ischemia/reperfusion led to the injury of myenteric neurons. From experiments *in vitro* and *in vivo*, we demonstrated that L-cysteine protected myenteric neurons from the injury. AOAA reversed the protective effect of L-cysteine. Also, L-cysteine played a protective role mainly by acting on intestinal macrophages via decreasing the expression of NLRP3, cleaved caspase-1, and mature IL-1β. L-cysteine increased cystathionine beta synthase and H_2_S produced by intestinal macrophages to protect myenteric mature neurons and enteric neural precursor cells from apoptosis. Moreover, the addition of IL-1β-neutralizing antibody alleviated the injury of myenteric neurons and enteric neural precursor cells caused by intestinal ischemia/reperfusion. Our study provided a new target for the protection of myenteric neurons in clinical intestinal ischemia/reperfusion injury.

## Graphical Abstract

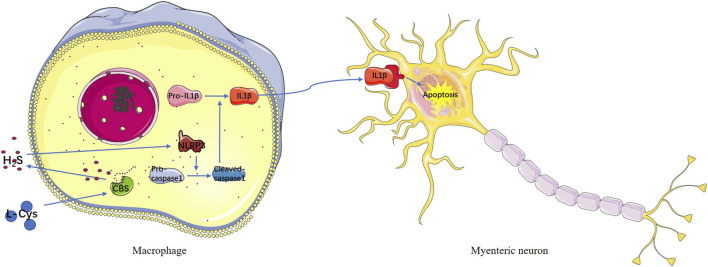

## 1 Introduction

Ischemia/reperfusion (IR) injury is a common pathophysiological process in clinic, which has a high incidence in severe trauma, shock, intestinal transplantation, and other surgical procedures (Mallick et al., 2004). The intestine is one of the sensitive organs to IR injury; intestinal ischemia leads to the increase in microvascular permeability and destruction of the mucosal barrier, and reperfusion itself also aggravates tissue damage, even causes systemic inflammatory reaction, and results in multiple organ dysfunction and death (Massberg and Messmer 1998; Jia et al., 2020). Intestinal neurons are particularly sensitive to IR injury. The digestive tract has a characteristic nervous system named the enteric nervous system (ENS). Enteric nerve plexus includes the submucous and myenteric nerve plexus. ENS has complete reflex pathways and regulates gastrointestinal movement, feeling, secretion, inflammatory reaction, and a variety of functions relatively independently. Meanwhile, ENS accepts the regulation of external nerve and the immune system. Based on the above, ENS is also called “gut–brain” (Gershon 2005; Mazzuoli-Weber and Schemann 2015). IR can lead to an irreversible loss of intestinal neurons and cause contraction and transport dysfunction of intestinal smooth muscle (Lindeström and Ekblad 2004; da Silva de Souza et al., 2015; Bistoletti et al., 2020), but the mechanism remains unclear. Although the regeneration of enteric neurons is highly controversial, more and more studies indicate that neurogenic cells exist in adult mice and human gut to maintain the number of enteric neurons, which express Nestin (Liu et al., 2009; Metzger et al., 2009; Kulkarni et al., 2017). These stem-cell-like neurons are called enteric neural precursor cells (ENPCs) and highly sensitive to inflammation, oxidative stress, or other stimulation.

Both innate and adaptive immune systems play important roles in IR injury (Zuidema and Zhang 2010; Eltzschig and Eckle 2011). More and more evidence has proved that macrophages are actively involved in IR injury. Macrophages can secrete a large number of pro-inflammatory or anti-inflammatory cytokines, which determine the prognosis of myocardial and renal IR injury (Hamacher-Brady et al., 2006; Lee et al., 2011). Yet, the mechanism of macrophages involved in intestinal IR injury remains obscure. There are a large number of macrophages existing in the mucosa, submucosa, and muscularis of the intestine. During intestinal IR injury, pro-inflammatory macrophages can be recruited and activated to produce a large number of inflammatory mediators such as IL-1β and IL-6, leading to neutrophil infiltration and aggravating intestinal mucosal injury (Liu et al., 2015). The mutual regulation between the ENS and the enteric immune system is the basis for the digestive tract to maintain homeostasis, and the mechanism involved remains uncharted (Yoo and Mazmanian 2017).

NLRP3 inflammasome belongs to the NLR protein family, a group of cytoplasmic protein complexes, widely existed in immune cells, including granulocytes, macrophages, and lymphocytes (Zhen and Zhang 2019). NLRP3 inflammasome can be activated by multiple stimuli, including a variety of microbial products, endogenous molecules, and particles (He et al., 2016). NLRP3 inflammasome activation leads to self-activation of the precursor caspase-1 to form cleaved caspase-1, which activates pro-IL-1β to produce activated mature IL-1β (Martinon et al., 2002). IL-1β is a potent pro-inflammatory molecule involved in a variety of immune diseases. IL-1β plays an important role in neuron injury of neurological diseases (Allan et al., 2005; Skelly et al., 2019). However, the underlying mechanism of NLRP3 inflammasome in myenteric neuron injury induced by intestinal IR has not been determined.

Since the occurrence of intestinal ischemia is inevitable, most studies have focused on drug therapy to improve reperfusion injury. L-cysteine (L-Cys) is a non-essential amino acid containing sulfur (Clemente Plaza et al., 2018). L-Cys, as the substrate of hydrogen sulfide (H_2_S), can be catalyzed by cystathionine beta synthase (CBS), gamma-cystathionase (CSE), and 3-mercaptopyruvate sulfurtransferase (3-MST) to produce H_2_S (Paul and Snyder 2015). As an important gas signal transmitter, H_2_S is involved in the regulation of many physiological and pathological processes, including inflammation, metabolism, neuroprotection, and vasodilation (Yang et al., 2008; Hu et al., 2011; Zhao et al., 2020). Saini et al. found that CBS and CSE were expressed in mouse RAW264.7 macrophage-like cell line and primary cultured peritoneal macrophages, and H_2_S produced by macrophages participated in the immune regulation against *Mycobacterium tuberculosis* (Saini et al., 2020). H_2_S could regulate the function of macrophages to participate in the sepsis, brain edema, transplant rejection, wound repair, and other pathophysiological processes (Miao et al., 2016; Zhuang et al., 2018; Zhou et al., 2019).

Based on the above findings, this study aimed to investigate whether macrophages are involved in myenteric neuron injury caused by intestinal IR, to elucidate the protective effect of L-Cys on the myenteric neurons after intestinal IR injury and its mechanism.

## 2 Materials and Methods

### 2.1 Reagents

L-Cysteine (L-Cys), aminooxyacetic acid (AOAA, CBS inhibitor), DL-propargylglycine (PAG, CSE inhibitor), lipopolysaccharide (LPS), and MCC950 (NLRP3 inflammasome inhibitor) were purchased from Sigma-Aldrich (St. Louis, MO). Control liposomes and clodronate liposomes were purchased from FormuMax (Silicon Valley, CA). Deoxyribonuclease Ⅰ was purchased from Beijing Solarbio Science and Technology Co. (Beijing, China). 2-Deoxy-D-glucose was purchased from J&K Scientific (Beijing, China). Armenian Hamster IgG Isotype Control and IL-1 beta Monoclonal Antibody were purchased from Invitrogen Life Technology (Foster City, CA). Antibodies for βⅢ-tubulin, Nestin, and IL-1β were purchased from Abcam (Cambridge, United Kingdom). Antibodies for cleaved caspase-1 and NLRP3 were purchased from Cell Signaling Technology (Danvers, MA). Monoclonal mouse anti-β-actin was purchased from Zhongshan Golden Bridge Biotechnology (Beijing, China). The secondary antibodies were purchased from Abcam (Cambridge, United Kingdom), Invitrogen Life Technology (Foster City, CA), and Zhongshan Golden Bridge Biotechnology (Beijing, China). Mouse ELISA kits were purchased from Dakewe Biotech (Shenzhen, China) and Boster Biological Technology (Wuhan, China). Dulbecco’s modified Eagle’s medium (DMEM), 10% heat-inactivated FBS, and 1% penicillin–streptomycin solutions were purchased from Gibco (Foster City, CA).

### 2.2 Experimental Animals

The mice used in this experiment were all male C57BL6/J mice aged 8–10 weeks, which were purchased from the Animal Center of Shandong University. Experimental mice were housed under pathogen-free conditions with a 12-h light–dark cycle in the animal care facilities. The suffering of the experimental animals was reduced to a minimum, and all animal experiments were approved by the Medical Ethics Committee for Experimental Animals, Shandong University School of Basic Medicine Sciences (ECSBMSSDU2020-2–006).

### 2.3 Intestinal Ischemia and Reperfusion

The mice were fasted overnight and anesthetized with sodium pentobarbital (50 mg/kg, intraperitoneally). The anesthetized mice were supine and fixed on the operating table. The intestinal IR model was established using a modified technique described previously (Chai et al., 2019). Briefly, the abdomen of mice was incised with a 1-cm midline laparotomy and the superior mesenteric artery (SMA) was isolated carefully. The SMA was occluded with a microvascular clamp for 45 min. The abdominal incision was closed temporarily and reopened to remove the clamp when the ischemia was completed. The reperfusion was performed for 4 h. The mice were randomly divided into four groups as follows: 1) the sham-operated group (Sham)—mice underwent the SMA isolation and were intraperitoneally (i.p.) injected with 0.2 ml normal saline (NS) after ischemia without SMA occlusion; 2) the intestinal IR group (IR)—the SMA was occluded, and 0.2 ml NS was i. p. injected after ischemia; 3) the L-Cys group (LC + IR)—the SMA was occluded, and 0.2 ml L-Cys (5 mg/kg) was i. p. injected after ischemia; and 4) the AOAA + L-Cys group (AOAA + LC + IR)—the mice were intraperitoneally injected with AOAA (50 mg/kg) 30 min before the operation, and then, the SMA was occluded, and 0.2 ml L-Cys (5 mg/kg) was i. p. injected after ischemia. The body temperature of the mice was kept at 37°C with a heating pad during the study period, and the mice were allowed to eat or drink after awakening from anesthesia. All the mice were euthanized, and samples were obtained for analysis after the 4-h reperfusion.

### 2.4 Myenteric Neuron Isolation and Culture

The isolation and culture of the myenteric neurons were performed according to the improved method in our previous study (Che et al., 2012; Gao et al., 2021). Briefly, the mice were anesthetized with sodium pentobarbital and a segment of 5-cm ileum was removed away from the terminal ileum. The cutoff ileum was quickly put in the ice-cold Krebs’ saline and slitted along the mesenteric junction with Venus scissors. The longitudinal muscle myenteric plexus (LMMP) was acquired by removing the mucosa, submucosa, serosal layer, and circular muscle layers in order. The pinned LMMP was digested with papain (10 mg/ml dissolved in Krebs’ saline) for 55 min at 37°C, and then, the LMMP was snipped into tiny pieces and removed from the digestion solution (1 mg/ml collagenase Ⅱ, 10% FBS, and 1% penicillin–streptomycin dissolved in DMEM) for 55 min at 37°C. After digestion, the cells were gathered by centrifugation for 8 min at 1000 RPM. After centrifugation, the supernatant was discarded and the cell mixture was gently resuspended with the complete medium. The cells were cultured in an incubator at 37°C with 5% CO_2_ for 6 days to get the myenteric neurons, and the medium was changed daily.

### 2.5 Macrophage Depletion

Clodronate liposomes (200 µL of the order preparation) were used to deplete macrophages in mice, and the control liposomes were used as a control as described in our previous experiments (Gao et al., 2021). Clodronate or control liposomes were i. p. injected the day before the establishment of the mouse intestinal IR model.

### 2.6 Cell Isolation and Culture

Ileac mononuclear cells (MCs) were isolated by the Percoll density gradient centrifugation using a modification of our previous procedure (Tang et al., 2019). The cell suspension was spun at 2000 RPM for 20 min in a 40–75% Percoll gradient after filtration to obtain ileac mononuclear cells. The cells at the interface were carefully collected for subsequent experiments. Murine peritoneal macrophages (PMs) were isolated from C57BL6/J mice by lavage of 3% fetal bovine serum (FBS) as previously described (Ray and Dittel 2010). PMs were obtained after concentration and cultured in a complete DMEM containing 10% heat-inactivated FBS and 1% penicillin–streptomycin in a humidified incubator at 37°C for subsequent experiments. RAW264.7 cells were obtained from the Cell Bank of Chinese Academy of Science (Shanghai, China) and cultured in complete DMEM containing 10% heat-inactivated FBS and 1% penicillin–streptomycin. Cells were treated with L-Cys (8 μM) for 30 min before LPS (100 ng/ml) was added in the presence or absence of AOAA (5 mM) or PAG (10 mM).

### 2.7 *In vitro* ATP Depletion and Hypoxia/Reoxygenation

In tissue ischemia, hypoxia and nutrient deficiency lead to a decrease in intracellular ATP levels, the extent of which varies with the severity and duration of ischemia (Atkinson et al., 2004). In *in vitro* cell experiments, the depletion of ATP and glucose was used to partially simulate ischemia and hypoxia *in vivo*. PMs were subjected to ATP depletion and HR as described previously (Lee et al., 2011). 2-Deoxy-D-glucose is a glucose analog that acts as a competitive inhibitor of glucose metabolism, inhibiting glycolysis via its actions on hexokinase, which can significantly reduce ATP levels in MCF-7 cells (Zhu et al., 2005). PMs were cultured in an airtight culture flask with glucose-free DMEM containing 2% FBS and 10 mM 2-deoxy-D-glucose with or without 100 ng/ml LPS in a humidified 5% CO_2_ incubator at 37°C for 45 min. After being washed twice with PBS, the medium was changed to DMEM containing 5% fetal calf serum and 1% penicillin–streptomycin with or without 8 μM L-Cys. Cells and the supernatant were collected separately for the next experiment after 4 h of continued incubation in a common culture flask.

### 2.8 Myenteric Neurons With Conditioned Medium

PMs were randomly divided into the control group and the HR group. Each group was divided into four subgroups as follows: 1) the control group—without any drug added; 2) the LPS group—100 ng/ml LPS was added for 45 min with or without ATP depletion and hypoxia; 3) the LC group—8 μM L-Cys was added for 4h (during reoxygenation in the HR group); and 4) the LPS-LC group—100 ng/ml LPS was added for 45 min with or without ATP depletion and hypoxia, and 8 μM L-Cys was added for 4h (during reoxygenation in the HR group). After incubation, the cell supernatant from each group was discarded, and the PMs were cultured in a complete DMEM for 24h after being washed twice with PBS. The supernatant from each group was collected as CM for the incubation of myenteric neurons for 48 h. The IgG isotype control (5 μg/ml) or IL-1β monoclonal antibody (5 μg/ml) was added to the CM to observe the role of IL-1β (González-Navajas et al., 2010). The number and the axon length of neurons were analyzed by immunofluorescence.

### 2.9 Measurement of H_2_S Concentration

A traditional methylene blue method was used to measure the concentration of H_2_S in cells as described in the previous method (Xin et al., 2018). A H_2_S detection kit (Jiancheng, Nanjing, China) was used, and the experiment was carried out according to the product instructions. Briefly, the supernatant of cultured cells or ileac MCs was collected and then incubated with zinc acetate to generate zinc sulfide, which could react with N,N-dimethyl-p-phenylenediamine sulfate (NNDPD) and generate methylene blue. Methylene blue has a maximum absorption peak at 665nm, and the concentration of H_2_S is calculated by measuring its absorbance. The absorbance value measured at 665 nm was denoted as A_1_, and the blank tube zero was denoted as A_0_, Δ A = A_1_-A_0_. The concentration of H_2_S was calculated according to the NaHS calibration curve ( $y=0.0044x,\;r^{2}=0.9988$ ) and formula ( $①\;H_{2}Sn\left(mol/mgprot\right)=\frac{\Delta A}{0.0044}\times V_{t}÷\left(V_{1}\times C_{pr}\right)\;②\;H_{2}S\left(nmol/ml\right)=\frac{\Delta A}{0.0044}\times V_{t}÷V_{1}$ ), where $V_{t}$ is the total reaction volume, $V_{1}$ is the sample volume in the reaction system, and *C*_pr_ is the protein concentration of the sample.

### 2.10 Immunofluorescence

The cells were fixed with 4% paraformaldehyde for 30 min at 25°C, and the tissue paraffin sections were dewaxed, then repaired in boiling sodium citrate buffer. The cells or paraffin sections were blocked with 10% donkey serum after washing. Antibodies against βⅢ-tubulin and Nestin were used for incubation. The images were observed by Olympus IX71.

### 2.11 Quantitative Real-Time PCR

The total RNA was extracted by the tissue/cell rapid extraction kit (SparkJade, Shanghai, China) and reverse-transcribed by a Takara PCR Thermal Cycler SP according to the product instructions. SYBR Premix (Takara Bio, Shiga, Japan) was used for real-time quantitative PCR. The primers were synthesized by Beijing Genomics Institute (Shenzhen, China), and their sequences are shown in Table 1.

**TABLE 1:** Primer pairs used for qRT-PCR.

Gene	Species	Forward primer	Reverse primer
β-actin	Mouse	GGC​TGT​ATT​CCC​CTC​CAT​CG	CCA​GTT​GGT​AAC​AAT​GCC​ATG​T
IL-1β	Mouse	GGC​AAC​CGT​ACC​TGA​ACC​CA	CCA​CGA​TGA​CCG​ACA​CCA​CC
IL-6	Mouse	TCC​TTC​CTA​CCC​CAA​TTC​CA	GTC​TTG​GTC​CTT​AGC​CAC​TCC
TNF-α	Mouse	CCC​TCA​CAC​TCA​GAT​CAT​CTT​CT	GCT​ACG​ACG​TGG​GCT​ACA​G
CBS	Mouse	GAA​GCC​TGG​AGA​CAC​TAT​CAT​T	CAT​CAC​GAT​AAT​GCA​GCG​ATA​G
NLRP3	Mouse	GCT​CCA​ACC​ATT​CTC​TGA​CCA	GGT​TGG​TTT​TGA​GCA​CAG​AGG

### 2.12 Enzyme-Linked Immunosorbent assay

The cell supernatant or tissue protein was collected after drug incubation or IR model treatment. According to the instruction, the specific protein concentration was detected by an ELISA precoating kit (Dakewe Biotech, Shenzhen, China).

### 2.13 Western Blotting Analysis

The protein from tissues or cells was extracted by RIPA lysis buffer (Boster Bio, Pleasanton, CA) and electrophoresed in SDS–polyacrylamide gel electrophoresis, and then transferred to PVDF membrane. The membranes were incubated with antibodies against IL-1β (1:1,000, Abcam), cleaved caspase-1 (1:1,000, CST), and NLRP3 (1:500, CST). *β*-Actin (1:1,000, Zhongshan Golden Bridge Biotechnology) was used as a housekeeping control, and the band intensities were quantified by the ImageJ software.

### 2.14 TUNEL Staining

For the quantitative study of neuronal apoptosis, the Apoptosis Detection Kit from Servicebio (Wuhan, China) was used according to the specification. The proportion of βIII-tubulin (red) and TUNEL (green) double-positive neurons (%) in every 10^4^ μm^2^ ganglion region was counted using the Image-Pro Plus software (Media Cybernetics, MD).

### 2.15 Organ Bath and Isometric Tension Recording

As described previously (Li et al., 2016), full-thickness ileal segments (1.5 cm long) were prepared and mounted longitudinally in 10-ml organ baths containing Krebs’ solution, which was maintained at 37°C by a thermostat pump. A resting tension of 1 g was applied, and the ileal segments were attached to an isometric force transducer (JH-2B; Chengdu Instrument Factory, Chengdu, China). The tension was recorded by a polygraph system (ML785-PowerLab, AD Instrument, Sydney, Australia). Tissues were allowed to equilibrate for at least 20 min, and the last 2-min data were used for analysis. The final data were expressed as the motility index (frequency of contractions × average contraction amplitude) to quantify changes in basal contractile activity as described previously (Rivera et al., 2012).

### 2.16 Statistical Analysis

All data were expressed as *mean ± SEM*. The grouping of the data was performed by *one-way ANOVA*. Statistical analysis was performed with the GraphPad Prism, version 5 (La Jolla, CA). *p* < 0.05 was considered statistically significant.

## 3 Results

### 3.1 L-Cys Alleviated the Injury of Myenteric Neurons and the Increase in Pro-inflammatory Factors Induced by Intestinal Ischemia/Reperfusion

**FIGURE 1:**
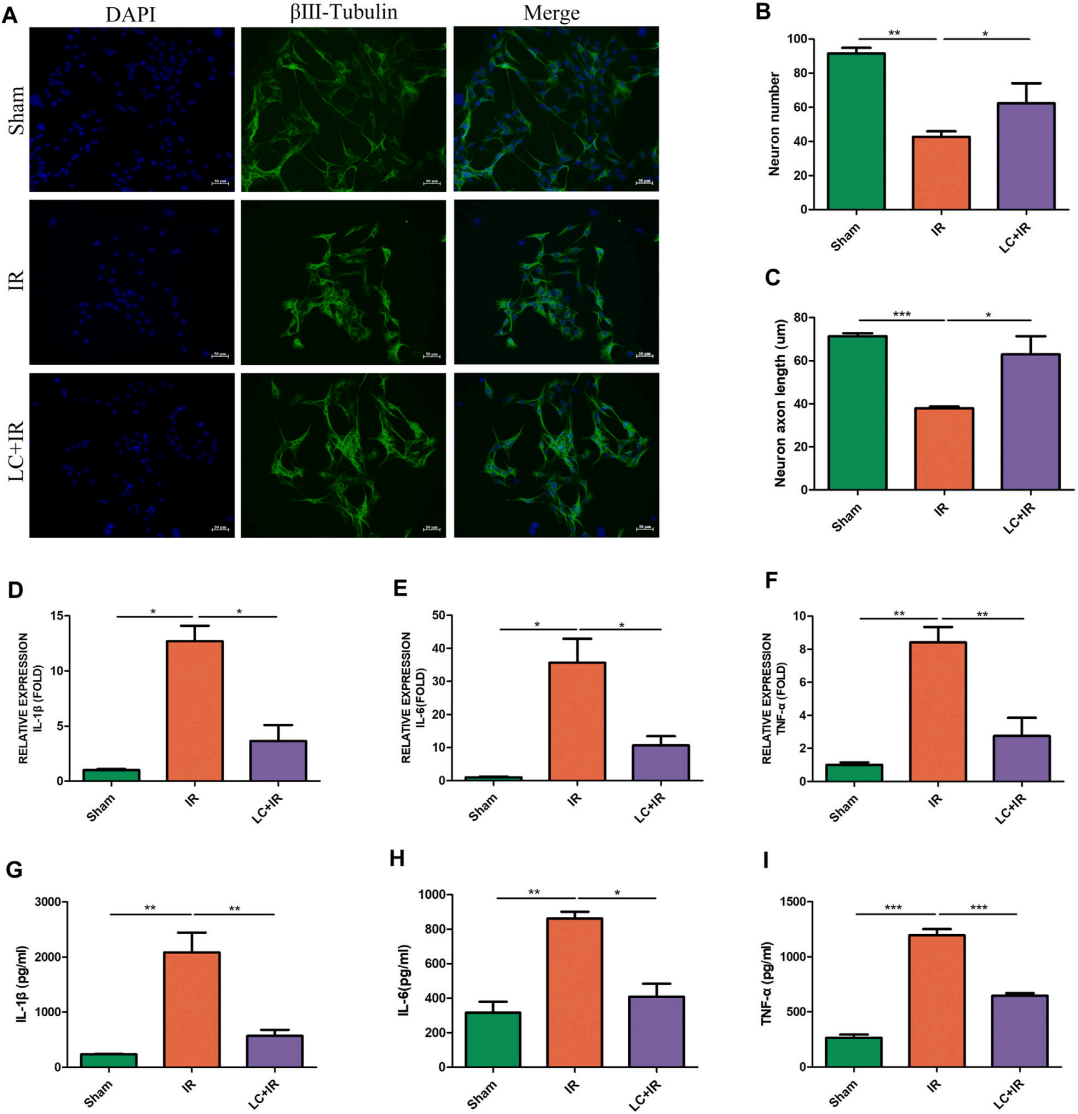
L-Cys alleviated the injury of myenteric neurons and the increase in pro-inflammatory factors induced by intestinal IR. After the IR model was performed, **(A)** intestinal myenteric neurons were isolated and cultured for 6 days *in vitro* for immunofluorescence staining with representative images of βⅢ-tubulin (green) and DAPI (blue). Scale bar = 50 μm. *N = 4*/group. **(B,C)** Quantitative analyses of the number of neurons and the length of axons. Six randomly captured images per animal were analyzed for each group. *N = 4*/group. **(D–F)** The mRNA levels of pro-inflammatory factors IL-1β, IL-6, and TNF-α in LMMP were detected by qRT-PCR. *N = 5*/group. **(G–I)** Protein levels of pro-inflammatory factors IL-1β, IL-6, and TNF-α in LMMP were detected by ELISA. *N = 4*/group. Values are *mean ± SEM*, *p < 0.05, **p < 0.01, and ***p < 0.001 according to ANOVA with the Newman–Keuls test.

After reperfusion, intestinal myenteric neurons were isolated and cultured *in vitro* for 6 days. Compared with the sham group, in the IR group, the number of neurons was reduced and the length of axons was shortened (Figures 1A–C). Compared with the IR group, in the L-Cys + IR group, the number of neurons was increased and the length of axons was also improved (Figures 1A–C). The cytokines in the intestinal LMMP were detected by qPCR and ELISA. We found that compared with the IR group, in the L-Cys + IR group, the mRNA and protein levels of pro-inflammatory cytokines IL-1β, IL-6, and TNF-α were reduced in different degrees (Figures 1D–I), among them the protein level of IL-1β decreased significantly (569.76 ± 109.30 pg/ml vs. 2085.88 ± 359.48 pg/ml; Figure 1G). These results suggested that intestinal IR led to myenteric neuron injury, accompanied by severe intestinal inflammation, which could be partly reversed by L-Cys.

### 3.2 Macrophages Were Involved in the Injury of Myenteric Neurons After Intestinal Ischemia/Reperfusion

**FIGURE 2:**
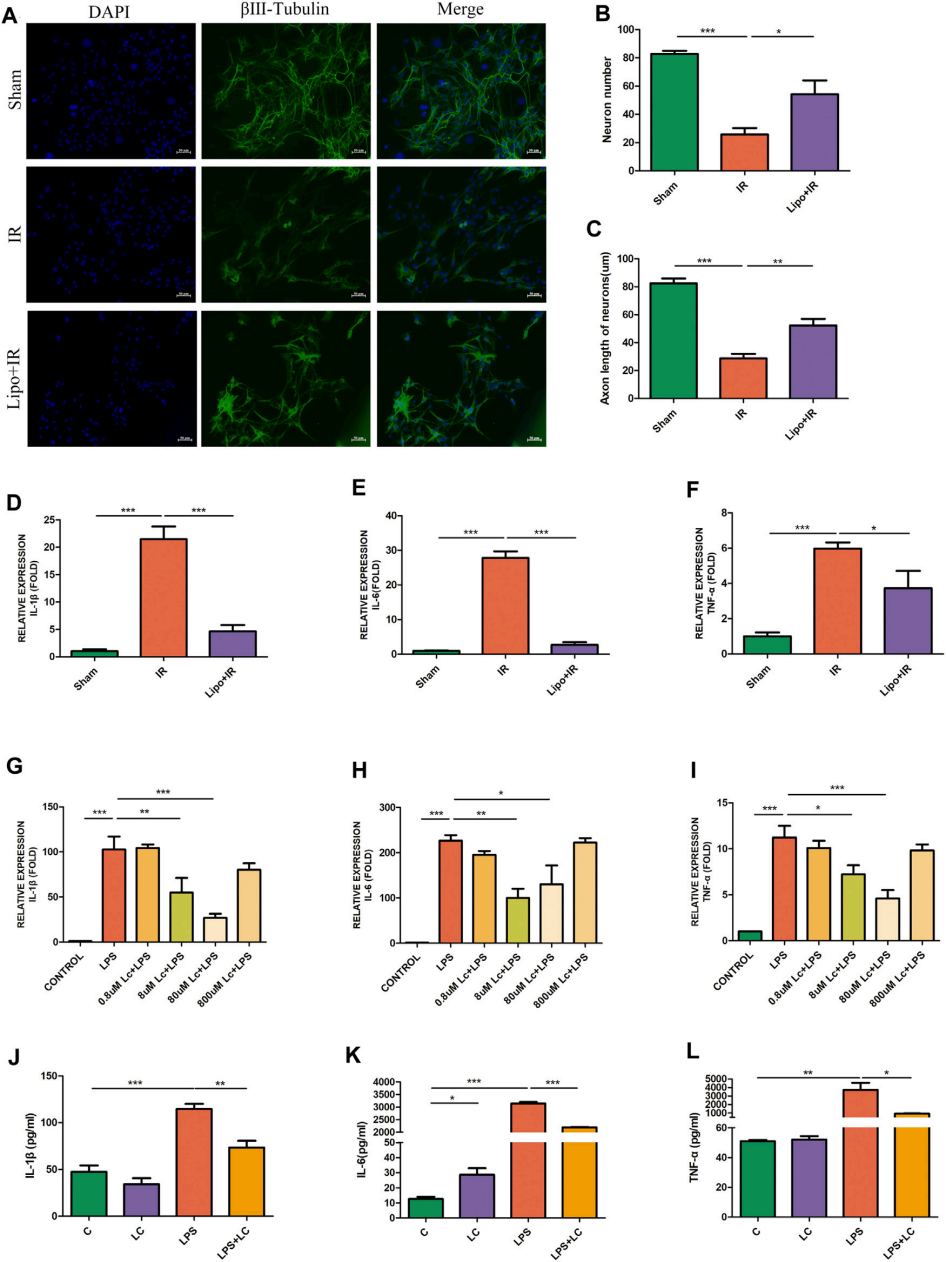
Macrophages were involved in the protection effect of L-Cys on the injury of myenteric neurons after intestinal IR. Clodronate or Control liposomes were i.p. injected the day before the establishment of the mouse intestinal IR model. **(A)** Representative photographs of immunofluorescent staining of βⅢ-tubulin (green) and DAPI (blue) among four groups. Scale bar = 50 μm. *N = 4*/group. **(B,C)** Quantitative analyses of the number of neurons and the length of axons. Six randomly captured images per animal were analyzed for each group. *N = 4*/group. **(D–F)** The mRNA levels of pro-inflammatory factors IL-1β, IL-6, and TNF-α in LMMP were detected by qRT-PCR. *N = 4*/group. **(G–I)** After L-Cys (from 0.8 to 800 μM) in coordination with LPS (100 ng/ml)-stimulated RAW264.7 cells, the mRNA levels of IL-1β, IL-6, and TNF-α were detected. *N = 4*/group. **(J–L)** Protein levels of pro-inflammatory factors IL-1β, IL-6, and TNF-α were detected by preincubating RAW264.7 cells with 8 μM L-Cys using ELISA. *N = 4*/group. Values are *mean ± SEM*, *p < 0.05, **p < 0.01, and ***p < 0.001 according to ANOVA with the Newman–Keuls test.

The intestine contains a large number of immune cells, among which macrophages are abundant and closely located with myenteric neurons (Gabanyi et al., 2016). The crosstalk between macrophages and neurons plays an important role in the maintenance of intestinal function (Muller et al., 2014). We used the clodronate liposomes (200 μL/mice, i. p.) to deplete peripheral macrophages to demonstrate the role of macrophages in intestinal IR injury. Compared with the IR group, in the Lipo + IR group, the decrease in the number of neurons was ameliorated and the length of axons was also increased (Figures 2A–C). Compared with the IR group, in the Lipo + IR group, the mRNA levels of pro-inflammatory factors IL-1β, IL-6, and TNF-α in LMMP were also decreased (Figures 2D–F). These results indicated that macrophages played an indispensable role in the myenteric neuron injury and the release of inflammatory factors induced by intestinal IR.

### 3.3 L-Cys Reduced Pro-inflammatory Factor Release in Lipopolysaccharide-Activated RAW264.7 Cells

The release of pro-inflammatory factors after intestinal IR was probably mediated by macrophages, and the alleviating effect of L-Cys on the increase in pro-inflammatory factors might also be the result of the effect of L-Cys on macrophages. Subsequently, we performed experiments with RAW264.7 cells *in vitro*. We firstly used four concentrations of L-Cys ranging from 0.8 to 800 μM to determine the optimal dosage of L-Cys in reducing the inflammation stimulated by LPS (100 ng/ml). In accordance with the principle of minimum effective drug concentration, we selected the suitable concentration of L-Cys as 8 μM according to the inhibition of IL-1β, IL-6, and TNF-α (Figures 2G–I). Next, we preincubated RAW264.7 cells with 8 μM L-Cys and then stimulated with pro-inflammatory macrophages with LPS. It was found that compared with the LPS group, in the L-Cys + LPS group, the expressions of IL-1β, IL-6, and TNF-α were all reduced (Figures 2J–L). Therefore, we proved that L-Cys alleviated the injury of myenteric neurons and the increase in pro-inflammatory factors after intestinal IR, which might be the result of L-Cys acting on macrophages.

### 3.4 L-Cys Attenuated the Production of Pro-inflammatory Factors in the Hypoxia/Reoxygenation Model of Peritoneal Macrophages *In Vitro*

**FIGURE 3:**
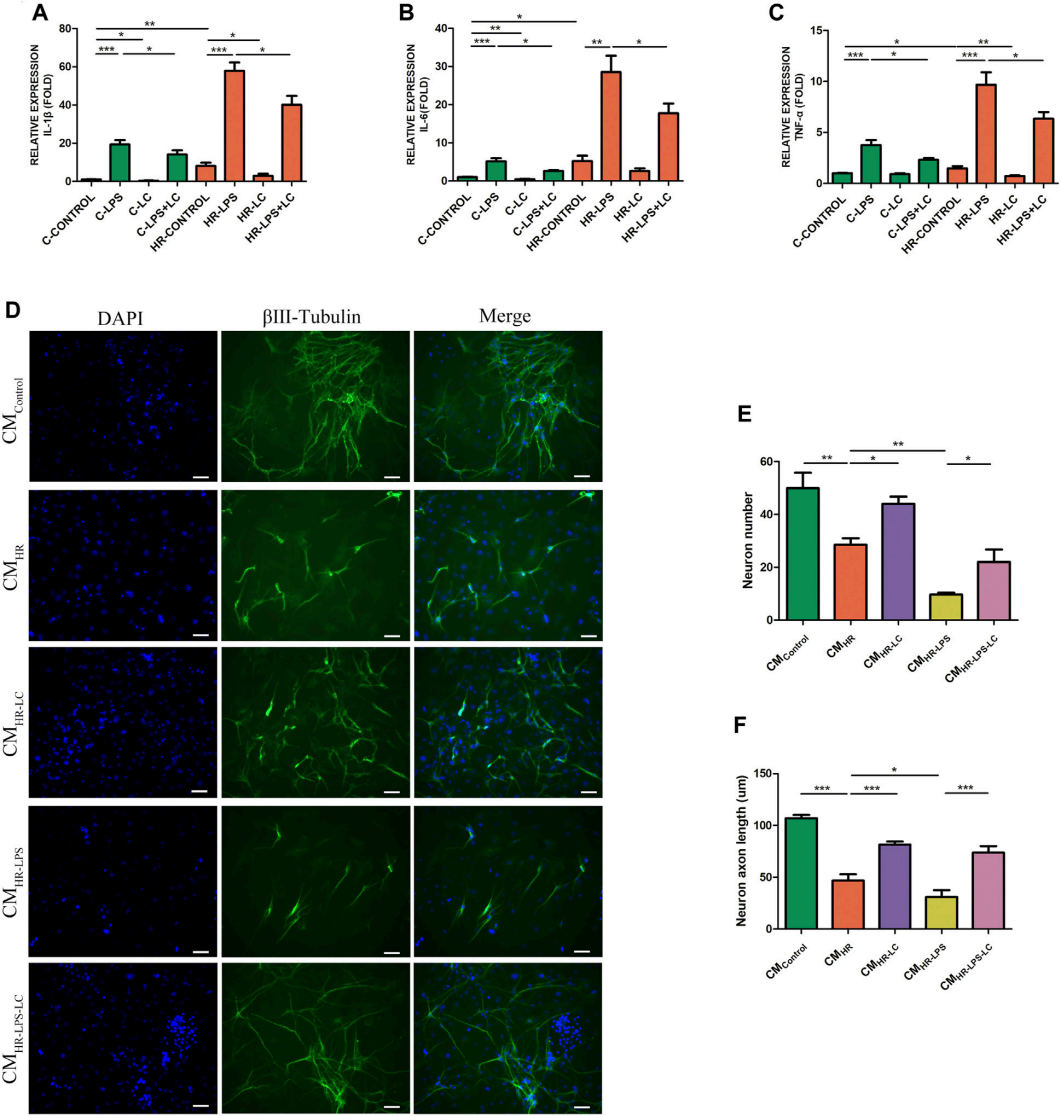
L-Cys attenuated the myenteric neuron injury and the production of pro-inflammatory factors in the HR model of PMs *in vitro*. (A–C) The mRNA levels of pro-inflammatory factors IL-1β, IL-6, and TNF-α of PMs were detected under normal and HR conditions by qRT-PCR. *N = 6*/group. Myenteric neurons were incubated with PM CM with or without the pretreatment of LPS and/or L-Cys under normal and HR conditions. **(D)** Representative photographs of immunofluorescent staining of βⅢ-tubulin (green) and DAPI (blue). Scale bar = 50 μm. *N = 4*/group. **(E,F)** Quantitative analyses of the number of neurons and the length of axons. Six randomly captured images per animal were analyzed for each group. *N = 4*/group. Values are *mean ± SEM*, *p < 0.05, **p < 0.01, and ***p < 0.001 according to ANOVA with the Newman–Keuls test.

ATP depletion and the HR model of PMs as described in the Methods section were established to simulate the IR process *in vitro*. The mRNA expressions of IL-1β, IL-6, and TNF-α were detected under normal and HR conditions separately. We found that L-Cys alleviated the increase in pro-inflammatory factors of PMs induced by LPS under both normal (C-LPS + LC) and HR (HR-LPS + LC) (Figures 3A–C) conditions. In addition, compared with the LPS group in the normal condition (C-LPS), HR cooperated with LPS (HR-LPS)-stimulated cells to produce more pro-inflammatory factors (Figures 3A–C). Interestingly, compared with the normal condition (C-CONTROL), PMs produced more pro-inflammatory cytokines only under the HR condition (HR-CONTROL), and the mRNA levels of IL-1β, IL-6, and TNF-α were all increased, and the increase in IL-1β was the most obvious (8.11 ± 1.71 vs. 1.00 ± 0.18; Figures 3A–C). These results indicated that macrophages indeed produced more pro-inflammatory factors after HR stimulation, and L-Cys played a mitigating role in advance, which was consistent with the results *in vivo*.

### 3.5 L-Cys Alleviated the Myenteric Neuron Injury Caused by the Supernatant of Peritoneal Macrophages After Hypoxia/Reoxygenation *In Vitro*

In order to verify whether pro-inflammatory cytokines produced by macrophages in IR led to myenteric neuron injury, and whether L-Cys had a protective effect on it, we conducted an *in vitro* experiment with a conditioned medium (CM). The myenteric neurons were incubated with PM CM with or without the pretreatment of L-Cys and/or LPS (Figure 3D). Compared with the control group (CM_Control_), the number of neurons and the length of axons decreased to some extent after incubation with H/R medium (CM_HR_, Figures 3E,F). Also, the decrease was more obvious after LPS incubation (CM_HR-LPS_, Figures 3E,F). Compared with the CM_HR_ group, the decrease in the number of neurons and the axon length was improved to some extent when preincubated with L-Cys (CM_HR-LC_; Figures 3E,F). Compared with the CM_HR-LPS_ group, the CM_HR-LPS-LC_ group had the same trend (Figures 3E,F). These results further verified that after intestinal IR, the pro-inflammatory factors produced by macrophages led to the injury of myenteric neurons, and such injury was improved using L-Cys ahead.

### 3.6 The Anti-inflammatory Effect of L-Cys Was Blocked by Aminooxyacetic Acid

**FIGURE 4:**
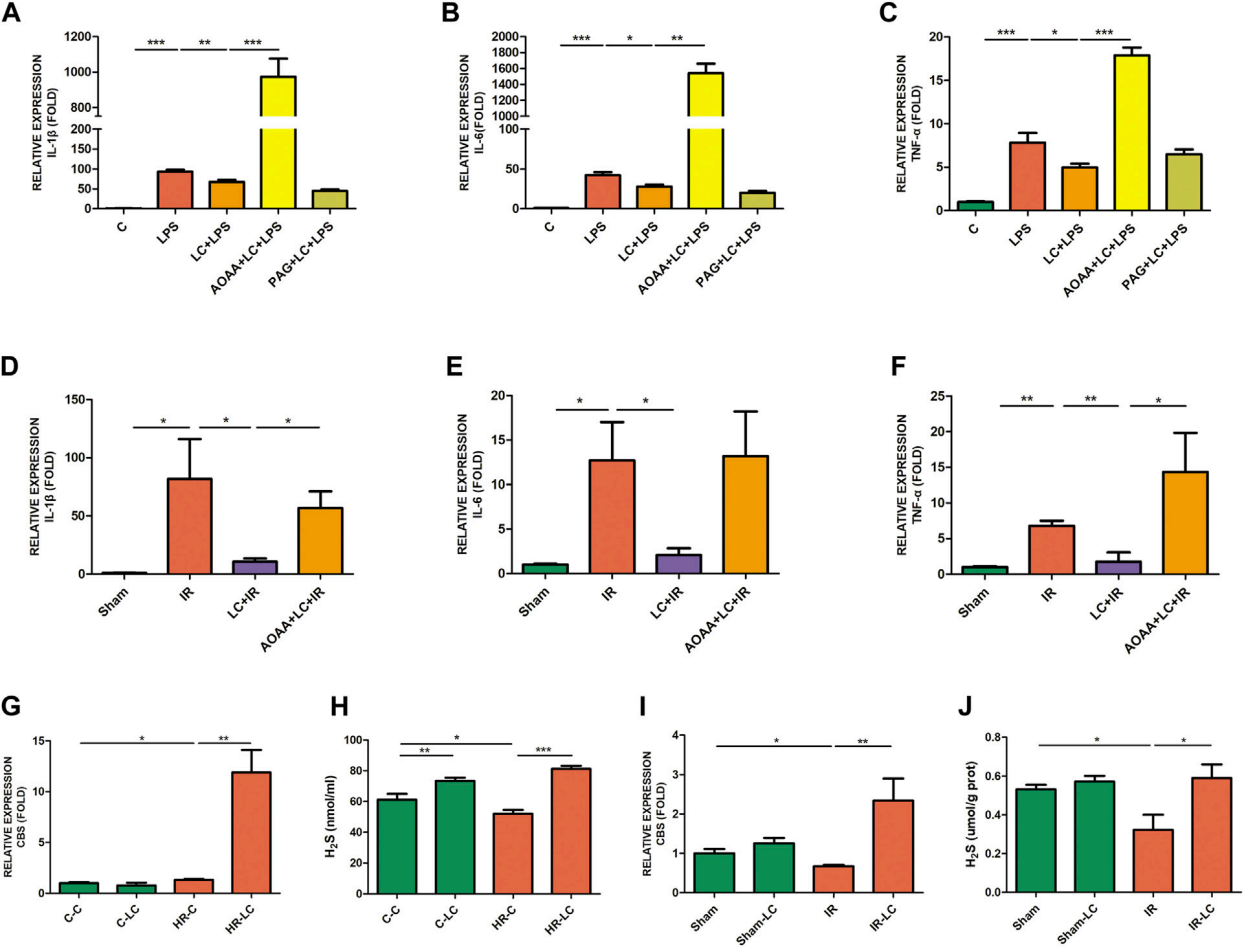
Decrease in L-Cys on pro-inflammatory factors was blocked by AOAA, and L-Cys up-regulated the CBS and H_2_S production. **(A–C)** The mRNA levels of pro-inflammatory factors IL-1β, IL-6, and TNF-α were detected through AOAA or PAG preincubation of RAW264.7 cells by qRT-PCR. *N = 4*/group. **(D–F)** After injecting AOAA into mice *in vivo*, the mRNA levels of pro-inflammatory factors IL-1β, IL-6, and TNF-α were detected by qRT-PCR. *N = 5*/group. **(G,I)** The mRNA levels of CBS in PMs or ileac MCs were detected under normal/Sham and HR/IR conditions by qRT-PCR. *N = 4*/group. **(H,J)** The H_2_S concentration was detected under normal/Sham and HR/IR conditions by a detection kit. *N = 6* or *4*/group. Values are *mean ± SEM*, *p < 0.05, **p < 0.01, and ***p < 0.001 according to *ANOVA with* the *Newman–Keuls* test*.*

As previously described, L-Cys preincubation reduced the production of pro-inflammatory factors in LPS-stimulated RAW264.7 cells. The mRNA levels of these pro-inflammatory cytokines were further detected by preincubating AOAA or PAG. We found that preincubation of AOAA reversed the protective effect of L-Cys on inhibiting IL-1β, IL-6, and TNF-α production, while PAG did not show the same effect (Figures 4A–C). Next, we detected the expression of these pro-inflammatory factors in LMMP after IR with or without AOAA injection. It was found that AOAA also reversed the effect of L-Cys on reducing the production of pro-inflammatory factors stimulated by IR *in vivo*, and the expression level of IL-1β changed most obviously (Figures 4D–F).

### 3.7 L-Cys Up-Regulated the Expression of Cystathionine Beta Synthase and H_2_S Production

The expression of CBS and H_2_S in ileac MCs or PMs was detected *in vivo* and *in vitro*. We found that compared with the HR-C or IR group, L-Cys increased the expression of CBS mRNA in PMs after HR (HR-LC) and in ileac MCs after IR (IR-LC) (Figures 4G,I). Compared with the control or sham group, the expression of CBS did not show a statistically significant increase in the L-Cys group (C-LC vs. C-C; Sham-LC vs. Sham; Figures 4G,I). At the meanwhile, the addition of L-Cys could increase the concentration of H_2_S under any condition (Figures 4H,J). Interestingly, compared with the control/sham group, the H_2_S concentration of macrophages was reduced in the HR or IR condition (HR-C vs. C-C; IR vs. Sham; Figures 4H,J). Through the above findings, we speculated that L-Cys reduced the production of pro-inflammatory factors and had a protective effect on IR injury, which was mainly due to the effect of L-Cys on the production of macrophage-derived H_2_S through CBS.

### 3.8 Neutralization of IL-1β Ameliorated the Myenteric Neuron Injury *In Vitro*

**FIGURE 5:**
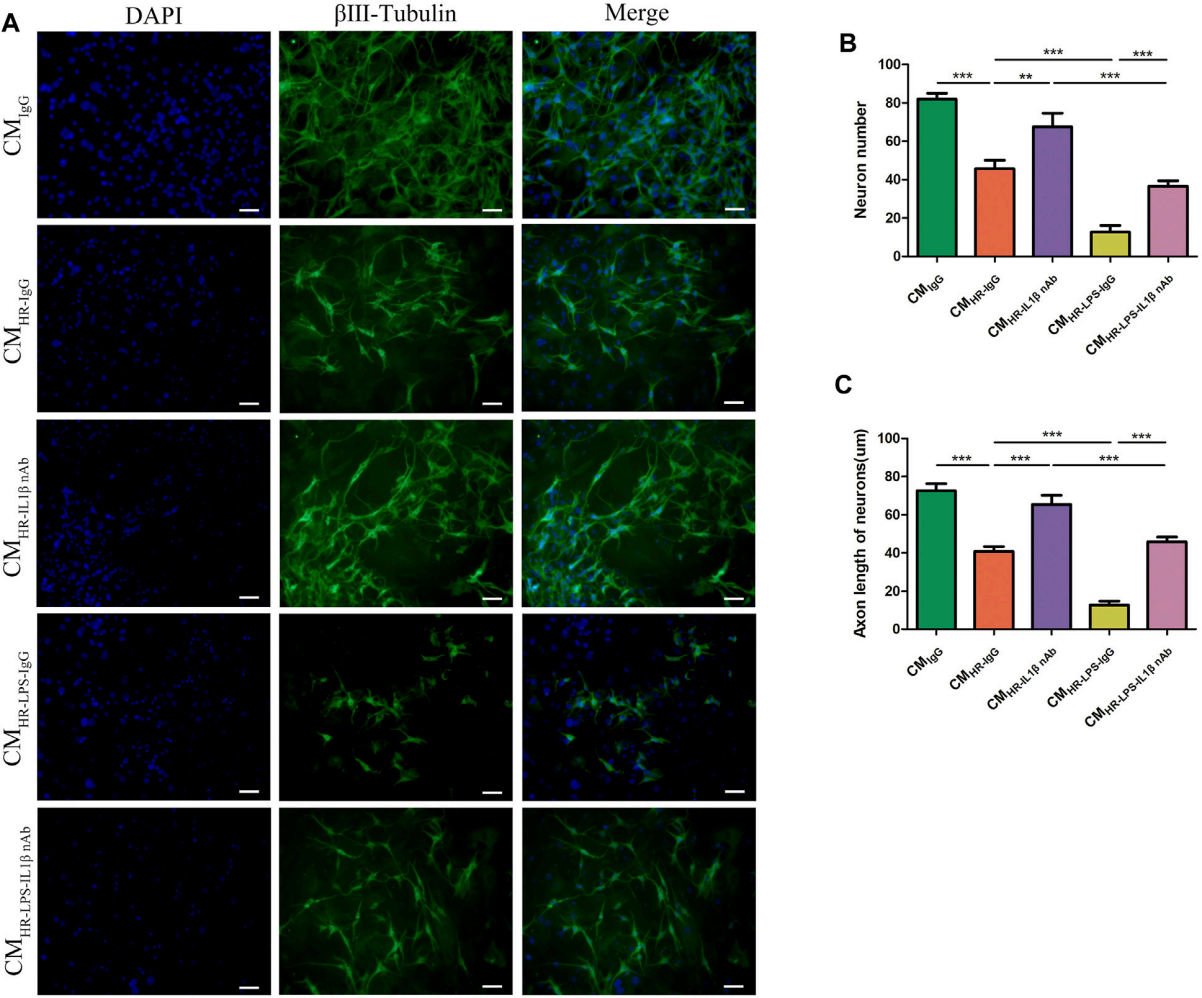
Neutralization of IL-1β ameliorated the myenteric neuron injury *in vitro*. Myenteric neurons were incubated with PM CM with or without the pretreatment of LPS and/or IL-1β-neutralizing antibody under normal and HR conditions. **(A)** Representative photographs of immunofluorescent staining of βⅢ-tubulin (green) and DAPI (blue). Scale bar = 50 μm. *N* = *5*/group. **(B,C)** Quantitative analyses of the number of neurons and the length of axons. Six randomly captured images per animal were analyzed for each group. *N = 5*/group. Values are *mean ± SEM*, *p < 0.05, **p < 0.01, and ***p < 0.001 according to ANOVA with the Newman–Keuls test.

Based on the above results, we found that the myenteric neuron injury might be caused by the increase in pro-inflammatory factors induced by IR, which had also been verified in the HR model *in vitro*. We also found that the pro-inflammatory factor IL-1β increased at a relatively higher level than others after HR/IR injury. Therefore, we employed a neutralizing antibody against IL-1β to clarify the role of IL-1β in the myenteric neuron injury caused by IR. *In vitro* ATP depletion and HR models as described above were established to simulate the IR environment. The myenteric neurons were incubated with different CMs with or without neutralizing antibody against IL-1β. Compared with the control group (CM_IgG_), the number of neurons and the length of axons decreased after incubation with HR CM (CM_HR-IgG_). Compared with the CM_HR-IgG_ group, the number of neurons and the length of axons recovered after adding IL-1β-neutralizing antibody (CM_HR-IL1β nAb_; Figures 5A–C). In addition, when the neurons were incubated with the CM from LPS-induced macrophages in the HR condition (CM_HR-LPS-IgG_), the greater injury was caused to neurons, and the addition of IL-1β-neutralizing antibodies (CM_HR-LPS-IL1β nAb_) also alleviated the neuron injury to some extent (Figures 5A–C). These results indicated that reducing IL-1β alleviated the myenteric neuron injury caused by HR.

### 3.9 L-Cys Inhibited IL-1β Release From Macrophages Caused by Ischemia/Reperfusion Through the NLRP3-IL-1β Pathway to Ameliorate Myenteric Neuron Injury

**FIGURE 6:**
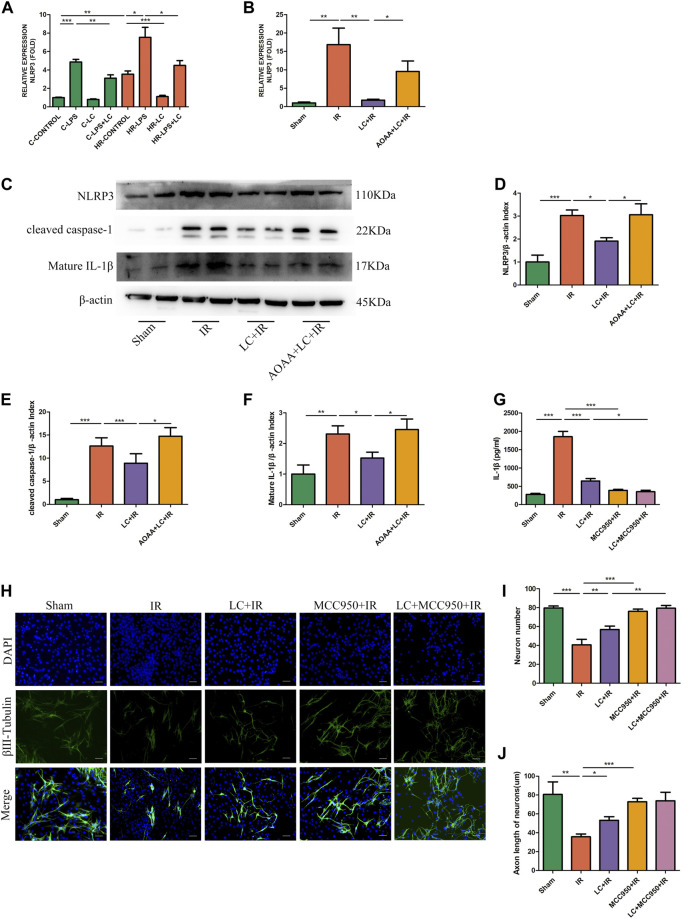
L-Cys ameliorated myenteric neuron injury caused by IR through the NLRP3-IL-1β pathway. **(A,B)** The mRNA levels of NLRP3 were detected under normal/Sham and HR/IR conditions by qRT-PCR. *N = 4*/group. **I** Representative immunoblots were detected for NLRP3, cleaved caspase-1, and mature IL-1β levels with Western blot analysis in each group. **(D–F)** Quantitative analyses of the relative levels of NLRP3/β-actin, cleaved caspase-1/β-actin, and mature IL-1β/β-actin in each group. *N = 6*/group. Values were normalized to *β*-actin. **(G)** Protein levels of IL-1β in LMMP were detected by ELISA. *N* = *4*/group. **(H)** After the IR model was performed, intestinal myenteric neurons were isolated and cultured for 6 days *in vitro* for immunofluorescence staining with representative images of βⅢ-tubulin (green) and DAPI (blue). Scale bar = 50 μm. *N = 4*/group. **(I,J)** Quantitative analyses of the number of neurons and the length of axons. Six randomly captured images per animal were analyzed for each group. *N = 4*/group. Values are *mean ± SEM*, *p < 0.05, **p < 0.01, and ***p < 0.001 according to ANOVA with the Newman–Keuls test.

From the above results, we knew that IL-1β played an indispensable role in the myenteric neuron injury caused by IR. Next, we investigated the potential mechanism through which L-Cys reduced IL-1β production from macrophages during IR. We examined the expression of NLRP3 at the mRNA level *in vitro* and *in vivo*. Compared with the C-CONTROL group, PMs expressed more NLRP3 during HR (HR-CONTROL; Figure 6A). Also, compared with the HR-CONTROL group, L-Cys alleviated the increase in NLRP3 caused by HR (HR-LC, Figure 6A). *In vivo*, L-Cys mitigated the increase in NLRP3 induced by IR in ileac MCs, and the addition of AOAA reversed this effect of L-Cys (Figure 6B). Then, we analyzed the expression of proteins associated with the NLRP3-IL-1β pathway by Western blot (Figure 6C). Compared with the sham group, in the IR group, the expression of NLRP3, cleaved caspase-1, and mature IL-1β was significantly increased (Figures 6D–F). Compared with the IR group, these proteins were decreased in the LC + IR group and this effect of L-Cys was blocked by AOAA (Figures 6D–F). In order to further verify the role of the NLRP3-IL-1β pathway in myenteric neuron injury caused by IR, we used NLRP3 inhibitor MCC950 for the next experiment. MCC950 is a highly specific small molecule inhibitor of NLRP3 inflammasome and is proven to inhibit canonical and noncanonical activation of NLRP3 inflammasome in many NLRP3-driven inflammatory diseases (Coll et al., 2015; Perera et al., 2018). After oral administration of MCC950 (20 mg/kg) 1 day before IR, the concentration of IL-1β in the intestinal LMMP was detected by ELISA (Figure 6G). We found that the level of IL-1β in either the L-Cys + IR or the MCC950 + IR group was significantly decreased compared with the IR group (Figure 6G). It was worth noting that compared with the L-Cys + IR group, the concentration of IL-1β in the MCC950 + IR group and L-Cys + MCC950 + IR was further reduced, but there was no significant difference between these two groups (Figure 6G). Correspondingly, the number of neurons and the length of axons were improved in the L-Cys + IR, MCC950 + IR, and L-Cys + MCC950 + IR groups, compared with the IR group (Figures 6I,J). At the meanwhile, compared with the L-Cys + IR group, the structure and number of neurons were better in the MCC950 + IR and L-Cys + MCC950 + IR groups (Figures 6I,J). From this, we drew the conclusion that NLRP3 inflammasome played an indispensable role in the production of IL-1β by macrophages during IR injury, and in the inhibition of the NLRP3-IL-1β pathway, which could relieve myenteric neuron injury caused by IR. L-Cys partly inhibited the NLRP3-IL-1β pathway of macrophages to reduce IL-1β production and protected myenteric neurons from IR injury.

### 3.10 L-Cys Decreased Apoptosis of Myenteric Neurons and Promoted Enteric Neural Precursor Cell Proliferation During Ischemia/Reperfusion

**FIGURE 7:**
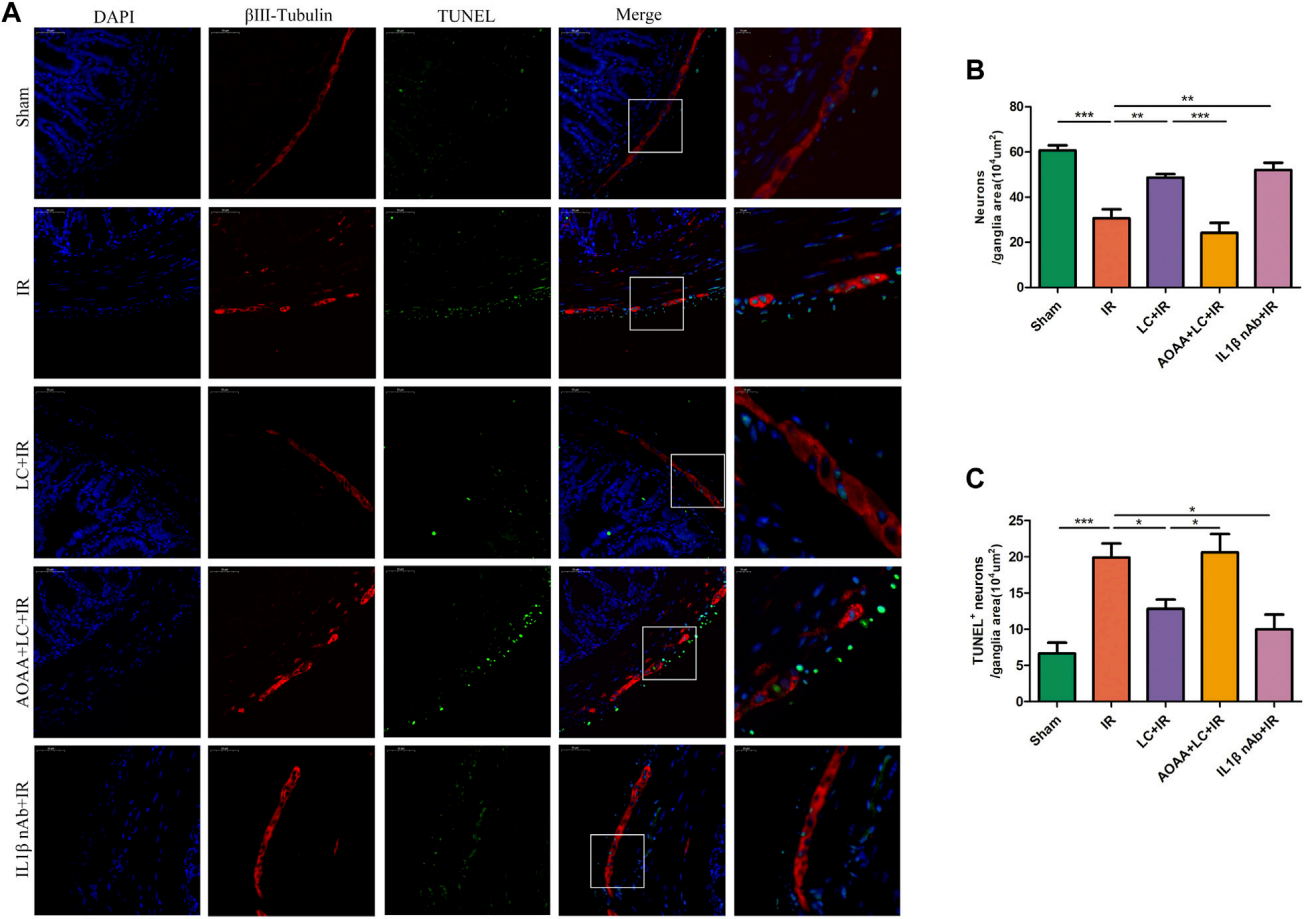
L-Cys decreased apoptosis of myenteric neurons caused by IR. **(A)** Representative photographs of immunofluorescent staining of TUNEL (green), βⅢ-tubulin (red), and DAPI (blue). Scale bar = 50 μm. *N = 4*/group. **(B,C)** Quantitative analyses of the number of neurons and TUNEL^+^ neurons in every 10^4^ μm^2^ ganglion region. Six randomly captured images per animal were analyzed for each group. *N = 4*/group. Values are *mean ± SEM*, *p < 0.05, **p < 0.01, and ***p < 0.001 according to ANOVA with the Newman–Keuls test.

**FIGURE 8:**
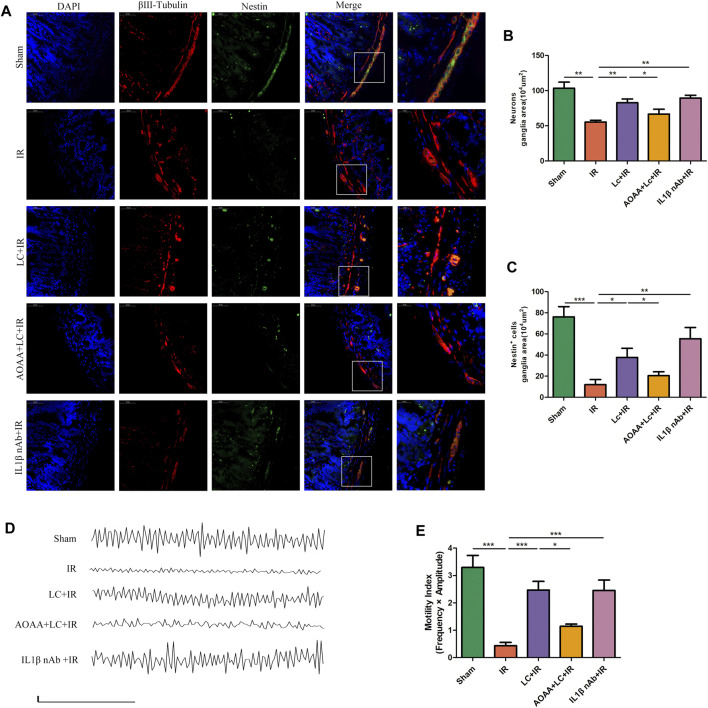
L-Cys protected Nestin^+^ ENPCs and improved intestinal contractile activity during IR. **(A)** Representative photographs of immunofluorescent staining of Nestin (green), βⅢ-tubulin (red), and DAPI (blue). Scale bar = 50 μm. *N = 4*/group. **(B,C)** Quantitative analyses of the number of neurons and Nestin^+^ neurons in every 10^4^ μm^2^ ganglion region Six randomly captured images per animal were analyzed for each group. *N = 4*/group. **(D)** Basal spontaneous activity of longitudinal muscle contractions from the mouse distal ileum (scale bars: 0.05g, 50s). *N = 5*/group. **(E)** Quantitative analyses of the motility index. *N = 5*/group. Values are *mean ± SEM*, *p < 0.05, **p < 0.01, and ***p < 0.001 according to ANOVA with the Newman–Keuls test.

To investigate the myenteric neuron injury induced by IR *in vivo*, we also detected the changes of neurons in intestine slices by immunofluorescence (Figure 7A). Compared with the IR group, in the LC + IR group the number of neurons in every 10^4^ μm^2^ ganglion region was significantly restored and AOAA reversed this effect of L-Cys (Figure 7B). Then, we detected the number of TUNEL^+^ myenteric neurons to observe the changes in apoptosis (Figure 7A). Compared with the sham group, in the IR group, the number of TUNEL^+^ neurons in every 10^4^ μm^2^ ganglion region was increased (Figure 7C). Compared with the IR group, in the LC + IR group, the number of TUNEL^+^ neurons in every 10^4^ μm^2^ ganglion region decreased significantly (Figure Figure7C). IL-1β-neutralizing antibody injection before IR significantly increased the number of neurons and decreased the number of TUNEL^+^ neurons (Figures 7B,C). The regeneration of enteric neurons is highly controversial. Recently, the Nestin^+^ ENPCs were come to the light and believed to rapidly proliferate and differentiate into newborn neurons to maintain the stability of the number of intestinal neurons (Almond et al., 2007). So, we detected the number of Nestin^+^ ENPCs in intestine slices during IR and observe the role of L-Cys in it (Figure 8A). Compared with the sham group, the number of total enteric neurons and Nestin^+^ neurons in every 10^4^ μm^2^ ganglion region decreased significantly during IR, and L-Cys ameliorated the loss of total neurons and Nestin^+^ neurons caused by IR (Figures 8B,C). AOAA partly eliminated the protective effect of L-Cys (Figures 8B,C). The i. p. injection of IL-1β-neutralizing antibody before IR significantly increased the number of total enteric neurons and Nestin^+^ neurons in every 10^4^ μm^2^ ganglion region (Figures 8B,C). These results proved that L-Cys alleviated the apoptosis of myenteric neurons and promoted neuron regeneration by inhibiting IL-1β release during IR. Abnormalities of the ENS can lead to intestinal motor dysfunction. We then performed organ bath experiments to detect contractions of the longitudinal muscle after IR and explore the role of L-Cys in it (Figure 8D). We used the motility index to quantify the basal contractile activity of the longitudinal muscle. After IR, the motility index was significantly reduced compared with the sham group (Figure 8E). L-Cys improved the motility index compared with the IR group, and this effect could be blocked by AOAA (Figure 8E). As same as the changes of enteric neurons during IR, IL-1β-neutralizing antibody injection before IR significantly improved the motility index of the longitudinal muscle (Figure 8E). These results indicated that L-Cys could improve intestinal motor dysfunction by protecting the enteric neurons from IR injury and IL-1β played an indispensable role in it.

## 4 Discussion

Our current research proved that intestinal IR led to the injury of ENS, which was mainly manifested as the reduction in the number of the mature neurons or ENPCs and the decrease in the length of axons leading to the dysfunction of enteric motility. Moreover, intestinal IR was accompanied by the increased expression of pro-inflammatory factors such as IL-1β, IL-6, and TNF-α, and the change in IL-1β was particularly obvious. The increase in these pro-inflammatory factors during IR injury was largely produced by macrophages in the intestine. Meanwhile, both *in vivo* and *in vitro* experiments confirmed that intestinal macrophages could synthesize H_2_S using L-Cys through CBS to reduce the production of pro-inflammatory factors during IR injury, so as to alleviate the injury of myenteric neurons including ENPCs. Moreover, the anti-inflammatory effect of L-Cys was mainly achieved through the NLRP3-IL-1β pathway. The protective effect of L-Cys was reversed when AOAA, a CBS blocker, was added. We proved for the first time that L-Cys increased the production of H_2_S by intestinal macrophages through CBS, and H_2_S acts on macrophages themselves in turn to reduce the secretion of pro-inflammatory factors and thus had a protective effect on the injury of myenteric mature neurons and especially ENPCs during intestinal IR. L-Cys reduced the production of IL-1β in macrophages, which provided the basis for clinical treatment of the injury of myenteric neurons and intestinal dyskinesia caused by intestinal IR.

Intestinal IR injury is common in clinical practice, and the mechanism is still complex. There are many researches about the mechanism, such as oxidative stress theory, inflammatory cascade theory, and microvascular theory (Kalogeris et al., 2016). The mechanism of damage to the ENS caused by IR remains unclear, and the effect of clinical drug treatment is not satisfactory. Therefore, it is urgent to find the changes in ENS and the underlying mechanism after intestinal IR. Our study initiatively demonstrated that intestinal IR led to the injury of myenteric neurons, which was manifested by a decreased number of neurons and shortened axon length. Also, the use of L-Cys before reperfusion alleviated the neuron injury. Studies have reported that intestinal IR injury is associated with the burst of pro-inflammatory cytokines, the production of reactive oxygen species (ROS), and the increase of nitric oxide (NO) levels (Liu et al., 2015; Filpa et al., 2017; Jia et al., 2020). There is growing evidence that neuron damage is closely associated with intestinal inflammation (Fricker et al., 2018; Hickman et al., 2018). We found that the expressions of IL-1β, IL-6, and TNF-α were increased to varying degrees after intestinal IR. Notably, the expression of IL-1β was relatively higher at the protein level. Interestingly, the use of L-Cys not only mitigated neuronal injury but also reduced pro-inflammatory cytokines in the intestine. This led us to hypothesize whether L-Cys reduced the expression of pro-inflammatory cytokines and thus protected myenteric neurons from IR injury.

A large number of studies have shown that macrophages play an irreplaceable role in the IR injury of the heart (Peet et al., 2020), liver (Ye et al., 2020), and kidney (Huen and Cantley 2017), but the role of macrophages in intestinal IR has been less studied. So, we used clodronate liposomes to deplete intestinal macrophages *in vivo* to confirm the role of macrophages during IR. This approach was well established and had been proven effective in a variety of models (Gliem et al., 2012; Yan et al., 2018; Gao et al., 2021). We found that the depletion of macrophages in mice before intestinal IR alleviated the injury of myenteric neurons and also reduced the increase in pro-inflammatory factors. This result provided the evidence for our hypothesis that L-Cys could protect intestinal neurons from IR injury by acting on macrophages. Therefore, we used RAW264.7 cells to verify *in vitro* and found that the preincubation of L-Cys inhibited the increase in pro-inflammatory cytokine production induced by LPS.

Since L-Cys depressed pro-inflammatory factors produced by LPS-activated RAW264.7 cells *in vitro*, we simulated the process of IR *in vitro* to observe the effect of L-Cys on PMs. Cell ATP depletion and the HR model were mature and widely used (Lee et al., 2011; Yu et al., 2018; Kormann et al., 2020). We found that L-Cys reduced the production of LPS-induced pro-inflammatory factors under both normal and HR conditions. Interestingly, PMs produced more pro-inflammatory cytokines during HR process even without LPS stimulation and L-Cys preincubation had an anti-inflammatory effect. This result was also consistent with that of our IR experiment *in vivo*. It was worth noting that the expression of IL-1β was also relatively higher in the detected pro-inflammatory factors, which was consistent with that *in vivo*. This result again confirmed our hypothesis that L-Cys alleviated the increase in pro-inflammatory factors released by intestinal macrophages during IR, especially IL-1β.

In order to demonstrate the protective effect of L-Cys on neurons *in vitro*, we directly observed neurons incubated with CM produced by PMs under different conditions. We found that the number of neurons and the length of axons decreased when the neurons were cultured with the medium of PMs stimulated by HR. Yet, when PMs were preincubated with L-Cys, the injury of neurons was alleviated. When the PMs were stimulated by both HR and LPS, L-Cys could partially alleviate the injury of neurons, but its protective effect on the number of neurons was weak. This *in vitro* result verified our hypothesis that L-Cys protected myenteric neurons via reducing the production of pro-inflammatory factors during intestinal IR.

L-Cys is the substrate of H_2_S. H_2_S is produced by three principal enzymes: CBS, CSE, and 3-MST (Dilek et al., 2020). H_2_S has a bidirectional effect on inflammation. Low concentration of H_2_S plays a regulatory role, including, in cell protection, and antioxidant and anti-inflammatory effects (Szabo and Papapetropoulos 2017). High concentration of H_2_S induces harmful effects in most cases, including pro-oxidation, cell inhibition, or cytotoxicity (Dilek et al., 2020). H_2_S plays an important role in many pathological conditions, including vascular disease, inflammation, reperfusion injury, nervous system diseases, and metabolic diseases (Dilek et al., 2020). H_2_S can inhibit neutrophil recruitment and inflammation (Wallace et al., 2010). H_2_S inhibits microglial overactivation and alleviates neurodegeneration and cognitive decline (Cao et al., 2018). H_2_S increases the proportion of myeloid-derived suppressor cells in circulation and alleviates ischemic symptoms (Wu et al., 2017). H_2_S inhibits the accumulation of CX3CR1^+^ cells and alleviates alcoholic steatohepatitis (Sutti et al., 2015). In most studies, exogenous administration of H_2_S plays an anti-inflammatory role in a variety of local and systemic inflammatory diseases. Because intestinal macrophages mainly expressed CBS and CSE, we used the inhibitors of both for verification. We found that the CBS inhibitor AOAA reversed the protective effect of L-Cys on the reduction in pro-inflammatory cytokines produced by LPS-stimulated cells. Yet, the CSE inhibitor PAG did not have such effect. Also, AOAA reversed the effect of L-Cys in reducing the increase in pro-inflammatory factors induced by IR *in vivo*. Next, we detected CBS expression and H_2_S concentration *in vitro* and *in vivo*. It was found that the CBS expression of PMs was slightly increased during the HR process compared with the control group. Yet, in *in vivo* experiment, the CBS expression of ileac MCs was decreased during IR injury. Moreover, under normal conditions, the addition of L-Cys did not produce statistically significant changes in the expression of CBS, while under HR or IR condition, the addition of L-Cys significantly increased the expression of CBS. We think it might be a self-protective response of the cells or the body to hypoxia stress. Compared with the control group, the HR or IR condition decreased H_2_S production. Also, L-Cys significantly increased the H_2_S concentration in any condition. From the above results, it was inferred that L-Cys acted on CBS in macrophages to increase the production of H_2_S during intestinal IR, and H_2_S acted on macrophages in turn to inhibit the secretion of pro-inflammatory factors, so as to reduce the pro-inflammatory factor production in the intestine to protect the neuron from IR injury.

Our study found that the expression of IL-1β was relatively higher compared with other inflammatory cytokines during the IR or HR condition. It also had been reported that IL-1β played an irreplaceable role in neuron injury (Xia et al., 1999; Allan et al., 2005; Pott Godoy et al., 2008; Skelly et al., 2019). Therefore, we used the neutralizing antibody of IL-1β to counteract the effect of IL-1β in order to verify the role of IL-1β produced by macrophages in neuron injury. Neurons were incubated with different CMs from PMs under different treatments. From this part of the results, we found that IL-1β was indeed one of the most obvious inflammatory mediators produced by macrophages for the injury of myenteric neurons during HR. NLRP3 inflammasome is composed of a sensor NLRP3 (NACHT, LRR, and PYD domain-containing protein 3), an adaptor ASC (apoptosis-associated speck-like protein), and a pro-caspase-1 (Hafner-Bratkovic et al., 2012). NLRP3 inflammasome can activate the precursor caspase-1, which forms the activated cleaved caspase-1; cleaved caspase-1 can activate pro-IL-1β to produce activated mature IL-1β (Martinon et al., 2002). Pro-caspase-1 is an inactive precursor protein in a resting state (Wang and Hauenstein 2020). During inflammasome assembly, pro-caspase-1 is recruited and drives its heterodimerization, autocleavage, and activation (Wang and Hauenstein 2020). IL-1β is the most potent pro-inflammatory factor released by the activation of the inflammasome, and it is implicated as the effector molecule in many NLRP3-driven diseases (Mangan et al., 2018). In the central nervous system, the significant elevation of IL-1β in the cerebrospinal fluid of brain injury, Alzheimer’s disease, and multiple sclerosis depends on the activation of NLRP3 within microglia (Wang and Hauenstein 2020). So, we investigated whether HR or IR could lead to NLRP3 activation in macrophages. First of all, *in vitro* studies found that the expression of NLRP3 in PMs increased during the HR condition, which could be restrained by L-Cys. In the intestinal IR model, L-Cys alleviated the increased expression of NLRP3, cleaved caspase-1, and mature IL-1β caused by IR, and the addition of AOAA reversed the protective effect of L-Cys. We also used the inhibitor of NLRP3 inflammasome, MCC950, to prove the effect of NLRP3-IL-1β pathway in the intestinal IR process. When MCC950 was added, the level of intestinal IL-1β was significantly decreased, and the injury to enteric neurons was relieved after IR. L-Cys had no obvious synergetic effect with MCC950 in the protection of enteric neurons during IR. These findings demonstrated that IR led to activation of NLRP3, which activated caspase-1 next and induced the increased production of IL-1β. L-Cys reduced the activation of NLRP3 and thus decreased the production of IL-1β. Extracellular ATP acting at the P2X purinoreceptor 7 (P2X7R) is taken for one of the most potent activators of the NLRP3 inflammasome (Franceschini et al., 2015). The P2X7R is mainly expressed on immune cells including macrophages (Tewari and Seth 2015). Recent studies have observed an increase in extracellular ATP release during IR injury such as renal or myocardial IR process. The increased extracellular ATP during IR binds to P2X7 receptor and activates NLRP3 inflammation to promote the production of cleaved IL-1β (Wu et al., 2020; Qian et al., 2021). Zhao et al. demonstrated endogenous H_2_S alleviated neuroinflammatory response in the pathogenesis of intracerebral hemorrhage by suppressing the ATP/P2X7R/NLRP3 pathway on microglia (Zhao et al., 2017). So, we are interested in the further exploration of whether P2X7R is involved in the regulation of L-Cys and macrophage NLRP3-IL-1β pathway during intestinal IR. We also observed the apoptosis of myenteric neurons by detecting TUNEL^+^ neurons. It was found that L-Cys alleviated the increase in TUNEL^+^ myenteric neurons induced by IR, and IL-1β had an indispensable role in the destruction of neurons during IR, which was also consistent with our previous results on the number of myenteric neurons. However, whether true stem-cell-like ENPCs exist in the gut remains highly controversial. More and more studies believed that there was a balance between neuron apoptosis and regeneration in the gut to maintain the number of enteric neurons (Almond et al., 2007). Kulkarni reported that approximately 31% of myenteric neurons were fated to programmed cell death in a 7-day period (Kulkarni et al., 2017). So, it was hard to explain how the numbers of enteric neurons remained remarkably constant for most of adult life without neurogenesis. In our study, we used Nestin antibody to label the ENPCs in order to observe the protective effect of L-Cys in neurogenesis during IR. We found that Nestin^+^ ENPCs were markedly reduced during IR. L-Cys could relieve the decrease in ENPCs and reduce the apoptosis of enteric neurons to maintain the number of enteric neurons constant. The injury to enteric neurons could cause dysfunction of intestinal motility. We also used the motility index to quantify the basal contractile activity of the intestinal smooth muscle. We found that L-Cys improved the intestinal motor dysfunction caused by IR, and this was largely because L-Cys reduced the level of IL-1β released by intestinal macrophages during IR.

In summary, the current findings demonstrated that L-Cys protected myenteric mature neurons and ENPCs from intestinal IR injury. Also, the CBS inhibitor, AOAA, reversed its protective effect. The protective effect of L-Cys was achieved by inhibiting the NLRP3-IL-1β pathway in intestinal macrophages. In a word, we proved the L-Cys induced intestinal macrophages to produce more H_2_S through increasing CBS expression and the increased H_2_S in turn acted on macrophages to inhibit the production of IL-1β via the NLRP3-IL-1β pathway to have a protective effect on myenteric neurons and intestinal motility during intestinal IR (Graphical Abstract). Our study provided a new target for the protection of myenteric neurons in clinical intestinal IR.

## Data Availability

The original contributions presented in the study are included in the article/Supplementary Material; further inquiries can be directed to the corresponding authors.

## Abbreviations

IR, ischemia/reperfusion; ENS, enteric nervous system; NLRP3, NOD-, LRR-, and pyrin domain-containing 3; L-Cys, L-cysteine; H_2_S, hydrogen sulfide; CBS, cystathionine beta synthase; CSE, gamma-cystathionase; 3-MST, 3-mercaptopyruvate sulfurtransferase; AOAA, aminooxyacetic acid; PAG, DL-propargylglycine; LPS, lipopolysaccharide; IL, interleukin; TNF-α, tumor necrosis factor-α; SMA, superior mesenteric artery; LMMP, longitudinal muscle myenteric plexus; DMEM, Dulbecco’s modified Eagle’s medium; MCs, mononuclear cells; PMs, peritoneal macrophages; HR, hypoxia/reoxygenation; CM, conditioned medium; ENPCs, enteric neural precursor cells.
